# Accumulation of eicosapolyenoic acids enhances sensitivity to abscisic acid and mitigates the effects of drought in transgenic *Arabidopsis thaliana*

**DOI:** 10.1093/jxb/eru031

**Published:** 2014-03-07

**Authors:** Xiaowei Yuan, Yaxiao Li, Shiyang Liu, Fei Xia, Xinzheng Li, Baoxiu Qi

**Affiliations:** ^1^College of Life Sciences, State Key Laboratory of Crop Biology, Shandong Agricultural University, Tai’an 271018, People’s Republic of China; ^2^Huasheng Agriculture Limited Liability Company, Qingzhou, Shandong 262500, People’s Republic of China; ^3^Department of Biology and Biochemistry, University of Bath, Claverton Down, Bath BA2 7AY,UK

**Keywords:** ABA, abscisic acid, *Arabidopsis*, drought resistance, eicosapolyenoic acids, IgASE1

## Abstract

IgASE1, a C_18_ Δ^9^-specific polyunsaturated fatty acid elongase from the marine microalga *Isochrysis galbana*, is able to convert linoleic acid and α-linolenic acid to eicosadienoic acid and eicosatrienoic acid in *Arabidopsis*. Eicosadienoic acid and eicosatrienoic acid are precursors of arachidonic acid, eicosapentaenoic acid, and docosahexaenoic acid, which are synthesized via the Δ^8^ desaturation biosynthetic pathways. This study shows that the *IgASE1*-expressing transgenic *Arabidopsis* exhibited altered morphology (decreased leaf area and biomass) and enhanced drought resistance compared to wild-type plants. The transgenic *Arabidopsis* were hypersensitive to abscisic acid (ABA) during seed germination, post-germination growth, and seedling development. They had elevated leaf ABA levels under well-watered and dehydrated conditions and their stomata were more sensitive to ABA. Exogenous application of eicosadienoic acid and eicosatrienoic acid can mimic ABA and drought responses in the wild type plants, similar to that found in the transgenic ones. The transcript levels of genes involved in the biosynthesis of ABA (*NCED3*, *ABA1*, *AAO3*) as well as other stress-related genes were upregulated in this transgenic line upon osmotic stress (300mM mannitol). Taken together, these results indicate that these two eicosapolyenoic acids or their derived metabolites can mitigate the effects of drought in transgenic *Arabidopsis*, at least in part, through the action of ABA.

## Introduction

Very-long-chain polyunsaturated fatty acids (VLCPUFAs), such as arachidonic acid (ARA; 20:4 Δ^5,8,11,14^) and eicosapentaenoic acid (20:5 Δ^5,8,11,14,17^), are key molecules that participate in various biological processes in the cell. In animals these VLCPUFAs affect health and nutrition by regulating the expression of genes through changes in the rate of transcription or post-transcriptional modifications (Schroeder *et al.*, 2008; Dimri *et al.*, 2010; Barnes *et al.*, 2012). These include neonatal retinal and brain development (Lauritzen *et al.*, 2001; Fleith and Clandinin, 2005; Leinster *et al.*, 2010) and cardiovascular health and disease prevention (Breslow, 2006; Von Schacky and Harris, 2007; Serini *et al.*, 2011). Since no higher plant can synthesize these VLCPUFAs naturally, genetic modification of plants to produce these health beneficial fatty acids has been the subject of intensive research in recent years. Consequently, various VCLPUFA-producing transgenic plants have been generated, including *Arabidopsis* (Qi *et al.*, 2004), linseed (Abbadi *et al.*, 2004), soybean (Kinney *et al.*, 2004), and *Brassica juncea* (Wu *et al.*, 2005; Cheng *et al.*, 2010; Venegas-Calerón *et al.*, 2010).

Although these VLCPUFAs are not commonly found in higher plants, they are abundant in lipids of pathogens seriously affecting crop yield, including *Phytophthora* species and related oomycetes (Sun *et al.*, 2013). These fatty acids are released into plant tissue from spores during early stages of infection (Choi *et al.*, 1992). Plants also respond to these fatty acids either by exogenous application or via pathogens containing them during infection, triggering the coordinated activation of defence-related responses (Bostock *et al.*, 1986). Studies show that ARA and eicosapentaenoic acid are potent elicitors of programmed cell death and defence responses in *Solanaceous* plants (Bostock *et al.*, 1981; García-Pineda *et al.*, 2004). Thus, these eicosapolyenoic acids can function as signalling molecules in various organisms (Rozhnova *et al.*, 2003; Savchenko *et al.*, 2010).

Taking advantage of eicosapolyenoic acid-producing transgenic *Arabidopsis* (Qi *et al.*, 2004), another study further validated the roles of ARA as a conserved signalling molecule in the regulation of biotic stresses in plants (Savchenko *et al.*, 2010). The expression of jasmonic acid-biosynthetic genes in the transgenic plants was upregulated, thus increasing their resistance to pathogenic microorganisms (Savchenko *et al.*, 2010). Therefore, eicosapolyenoic acids, such as ARA, play a regulatory role as novel pathogen-associated molecular patterns (or PAMPs) in oomycete-plant defence signalling networks (Bostock *et al.*, 2011).

In plants, it is well recognized that C_16_ and C_18_ polyunsaturated fatty acids such as hexadecatrienoic acid (16:3 Δ^7,10,13^) and α-linolenic acid (ALA; 18:3 Δ^9,12,15^) as well as their derived metabolites can modulate signal transduction pathways evoked by abiotic stress (Zhang *et al.*, 2005; Torres-Franklin *et al.*, 2009). This is because these fatty acids could reduce the structural and/or functional damages of cellular membranes caused by physiological stresses (Somerville and Browse, 1991; Allakhverdiev *et al.*, 2001; Iba, 2002; Upchurch, 2008). However, currently there are no reports on the roles that eicosapolyenoic acids, or indeed any other C_20_+ VLCPUFAs, play in abiotic stress responses in plants. Therefore, there is an urgent need to investigate the effect of these novel fatty acids on crop physiology and adaptation to the environment before introducing VLCPUFA-producing transgenic crops into the field for commercial production.

We generated transgenic *Arabidopsis* plant lines that produced appreciable amounts of the eicosapolyenoic acids eicosadienoic acid (EDA; 20:2 Δ^11,14^) and eicosatrienoic acid (ETrA; 20:3 Δ^11,14,17^) (Fraser *et al.*, 2004). This was achieved by constitutive expression of a Δ^9^ elongase gene, *IgASE1*, from the docosahexaenoic acid-producing marine microalga *Isochrysis galbana* (Qi *et al.*, 2002, 2003). Analysis of the fatty acids in the leaf glycerolipids revealed that these two novel fatty acids were particularly rich in membrane phospholipids such as phosphatidylcholine, phosphatidylethanolamine, phosphatidate, and phosphatidylinositol (Fraser *et al.*, 2004). In this study, we isolated a higher EDA- and ETrA-producing transgenic line where these two fatty acids constituted approximately 25 mol% of total leaf fatty acids. In contrast to the previous lower-producing (15%) line, it exhibited some abnormal morphology coupled with enhanced drought resistance. As a stress signal, abscisic acid (ABA) plays important roles in regulating drought response in plants (Ashraf, 2010). Therefore, we monitored the transcript levels of genes involved in the biosynthesis of ABA as well as other stress-related genes in this transgenic line and found that the drought resistance of the transgenic *Arabidopsis* was both ABA-dependent and -independent.

## Materials and methods

### Plant material and growth conditions

*Arabidopsis thaliana* ecotype Columbia 4 (Col-4) and *35S:IgASE1* transgenic plants in the Col-4 background (Qi *et al.*, 2004) were used for this study. Seed germination and plant growth conditions were as according to Pandey *et al.* (2006). Briefly, seed batches that were produced, harvested, and stored under identical conditions for 2 months were used. About 60 seeds from the wild-type (WT) and transgenic plants were surface sterilized and plated on the same petri dish containing 0.8% agar, ½ Murashige and Skoog (MS) salts, and 1% (w/v) sucrose. They were either chilled at 4 °C in the dark for 48h (stratified) or moved directly (non-stratified) to an environmental controlled growth room to germinate under a 16/8h light/dark regime at 21±1 °C. For soil-grown plants, 7-day-old plate-grown seedlings were transferred to 4×4cm pots filled with Levington F_2_ compost and cultivated under the same long day conditions. After 4 weeks the leaf areas of four individual plants were measured. For drought-stress assays, plants were grown in 6×6cm pots under 10/14h light/dark at 21±1 °C. After 3 weeks, water was withheld for 22 days and then pots were subsequently drenched with water.

For ABA and glycerol treatments, indicated concentrations of ABA and 2% glycerol were included in the ½ MS medium. To study the effect of EDA and ETrA, seeds were germinated on the same media combination that also contained the indicated amount of EDA, ETrA, or EDA+ETrA (sodium salt; Nu-Chek Prep, Elysian, MN, USA). Both EDA and ETrA were dissolved in 1% NP40 (Sigma-Aldrich, St Louis, MO, USA) to make a stock solution of 2.5mM. Germination is defined here as an emergence of the radicle through the seed coat. To study the response of dehydration by mannitol and glycerol, 3-day-old ½ MS-grown seedlings were transferred to fresh ½ MS, ½ MS containing 300mM mannitol, or 3% (v/v) glycerol and grown for further 7 days. The experiments were repeated three times. The data shown are means ± SE of all three experiments, unless stated otherwise.

### Fatty acid analysis

Fatty acids from leaves were extracted and converted to their fatty acid methyl esters as described by Browse *et al.* (1986). The fatty acid methyl esters were analysed by gas chromatography on a 30 m × 0.25mm DB-23 column (J&W Scientiﬁc, Folsom, CA, USA) using heptadecanoic acid (17:0) as an internal standard and quantiﬁed by ﬂame ionization detection (Fraser *et al.*, 2004).

### Stomatal aperture assay

Stomatal aperture was measured according to Pei *et al.* (1997) with slight modifications. Briefly, 10 of the first fully expanded rosette leaves were carefully removed from 4-week-old soil-grown plants cultivated under long days as described above. They were floated on buffer containing 10mM 2-(*N*-morpholine)-ethanesulphonic acid (MES), 50mM KCl, and 0.1mM CaCl_2_ (pH 6.15) and exposed to cool white light (120 µm·m^−2^·s^−l^) at 21 °C for 2h to induce stomatal opening. They were then transferred to the same buffer, with or without 10 μM ABA, and incubated for a further 2h. Abaxial epidermal peels were carefully removed and observed in the same buffers with an Olympus BX51 light microscope using a 20× objective lens. Images were captured using an Olympus DP71 U-TV0.5XC-3 digital camera attached to the microscope. Widths and lengths of approximately 60 stomata were measured that had guard cells with an inner edge of 16–22 µm (Pei *et al.*, 1997), and estimated using Image J software. Data are the means of three experiments.

### ABA assay

Leaves were harvested from 4-week-old WT and transgenic plants. Both fresh and dehydrated leaves (air-dried for 1h) were immediately frozen in liquid nitrogen. They were ground to a fine powder and homogenized in 90% (v/v) methanol containing 200 mg·l^−1^ diethydithiocarbamic acid sodium salt. The extracts were then incubated in a capped glass tube in darkness at 4 °C for 16h, followed by low-speed centrifugation at 2000rpm for 5min. The methanolic supernatant was recovered, dried, and the residue dissolved using methanolic Tris buffer (10% methanol/50mM Tris, pH 8.0/1mM MgCl_2_/150mM NaCl). An ABA ELISA quantitation kit was used to determine ABA content following the manufacturer’s instructions (Agdia, Elkhart, IN, USA; http://www.agdia.com).

### Water loss from detached leaves

Five leaves of similar developmental stages from 4-week-old soil-grown plants were detached and weighed immediately. They were placed on a laboratory bench and the weight of individual leaves recorded every hour for 7h. The relative fresh weight at each time was calculated as the percentage of the initial fresh weight to indicate the rate of water loss from the leaves. The experiment was repeated three times.

### RNA isolation and real-time PCR

Seven-day-old seedlings were transferred to liquid MS medium supplemented with 100 μM ABA, 300mM mannitol, or solvent control and incubated for 3h with gentle shaking. After this treatment seedlings were frozen in liquid nitrogen and ground into a fine powder. Total RNA was isolated from the frozen tissue using Trizol reagent (Transgen Biotech, Beijing, China) following the manufacturer’s instructions. RNA was further purified using the RNAeasy mini kit (Qiagen, Valencia, CA, USA) and 1 μg was used for the synthesis of first-strand cDNA using the SuperScript First-Strand Synthesis System (Life Technologies, Carlsbad, CA, USA). Real-time quantitative PCR was performed using gene-specific primers (Supplementary Table S1 available at *JXB* online) and TranStart Green qPCR SuperMix (Transgen Biotech). *Actin2* gene (Yoo *et al.*, 2010) was used as an internal normalization control. Fold change in gene expression was calculated using ΔCt values according to Schmittgen *et al.* (2008).

### Statistical analysis

All data presented are from at least three replicate experiments. Mean values and standard errors of the means were calculated, and the significance of differences was evaluated by Student’s *t* test. One-way analysis of variance was used to evaluate significant differences between multiple treatments.

## Results

### Identification and characterization of the *IgASE1* elongase gene in *Arabidopsis*

**Fig. 1.**
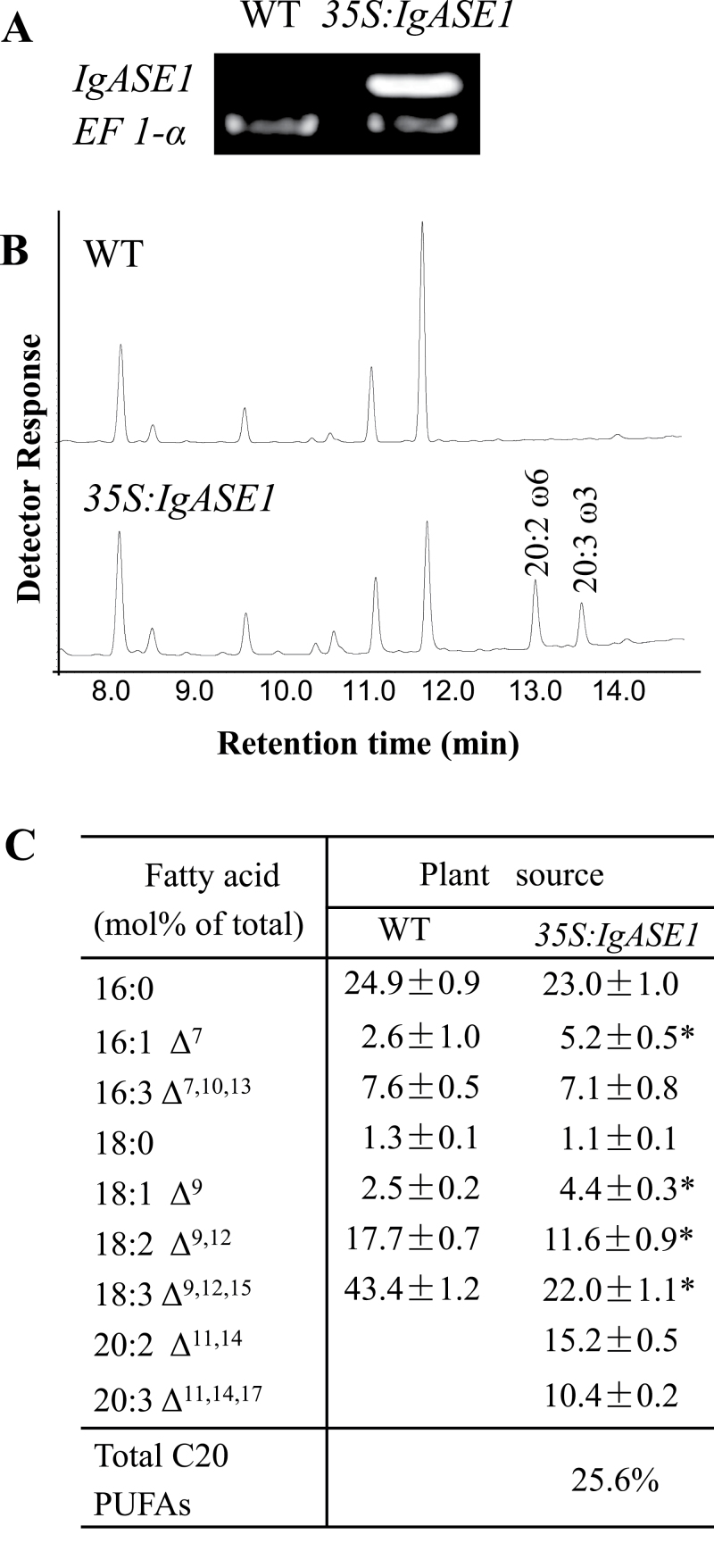
Expression of *IgASE1* in *Arabidopsis* leads to production of EDA and ETrA. (A) RT-PCR analysis shows expression of the *IgASE1* in transgenic *Arabidopsis*. PCR reactions were performed to amplify full-length transcript of *IgASE1*; *EF1-1-α* was used as a control. Samples were taken for analysis after 28 PCR cycles. (B) GC profiles of *A. thaliana* leaf fatty acid methyl esters. (C) Fatty acid composition. Total fatty acids were extracted from leaves of WT or transgenic plants. *P < 0.05 in a Student’s *t* test.

Homozygous single-copy transgenic *Arabidopsis* plants expressing *IgASE1* were identified based on herbicide resistance. RT-PCR was used to detect the transcript level of *IgASE1* from rosette leaves of 4-week-old plants and to show that *IgASE1* was indeed expressed in these transgenic plants (Fig. 1A). Total fatty acid content in leaf tissue was measured by gas chromatography. Transgenic plants contained two additional fatty acids compared to the WT (Fig. 1B). These were previously identified as EDA and ETrA and considered to be the elongation products of the IgASE1 elongase component (Fraser *et al.*, 2004). In one of the highest producers, these two fatty acids accumulated up to 15.2 and 10.4 mol% of total fatty acid, representing conversions of 56.7 and 32.1% of their precursor C_18_ substrates linoleic acid (LA) and ALA, respectively. This coincided with a significant decrease in LA and ALA and a small increase in 16:1 and 18:1 (Fig. 1C).

### *35S:IgASE1* transgenic *Arabidopsis* exhibited altered morphology

**Fig. 2.**
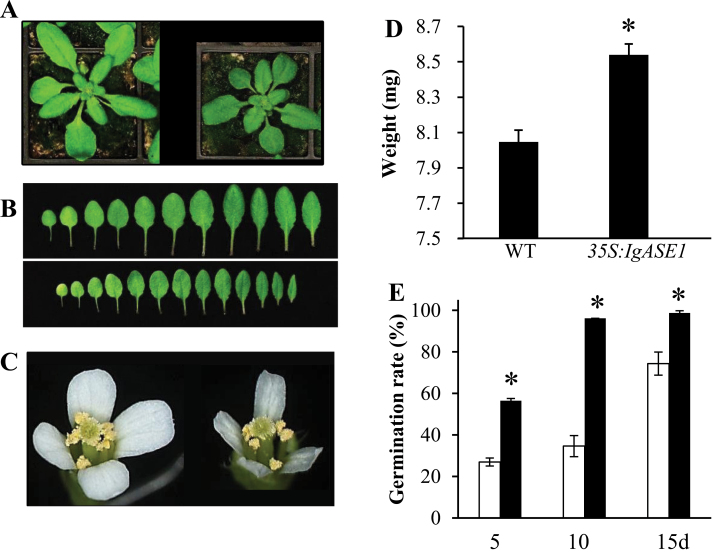
Phenotypic comparison of WT and *35S:IgASE1* transgenic *Arabidopsis* plants. (A) Three-week-old WT (left) and transgenic (right) plants. (B) Comparison of rosette leaves from WT (top) and transgenic (bottom) plants. (C) Flowers from WT (left) and transgenic (right) plants. (D) Dry weight of 500 seeds of WT and transgenic *Arabidopsis*. The experiment was repeated three times and the data were averaged; values are means ± SE; *n* = 500 for each experiment. (E) Percentage germination of seeds of WT (white columns) and transgenic (black columns) plants that were dried for 5, 10, and 15 days after harvest; 56 seeds were germinated on ½ MS. Values are means ± SE from three repeats. *P < 0.05 in a Student’s *t* test. This figure is available in colour at *JXB* online.

Although there were no significant phenotypic differences between WT plants and a previously identified transgenic *Arabidopsis* plant line in which the sum of the two fatty acid products accounted for about 15 mol% of the total fatty acids (Fraser *et al.*, 2004), the newly identified high-EDA- and -ETrA-producing transgenic plant line exhibited some phenotypic differences compared to the WT *Arabidopsis*. At the rosette stage, transgenic leaves were smaller, rounder, and a darker green than WT leaves. Total rosette leaf area of the transgenic lines was 4.3±0.5cm^2^ (*n* = 4) compared to 6.9±0.6cm^2^ (*n* = 4) for WT plants, leading to an overall smaller plant stature (Fig. 2A, B). The flowers of the transgenic plants were smaller with smaller petals that appeared translucent and rarely opened fully during the early flowering stages (Fig. 2C). Although no significant differences in seed phenotype were apparent, the mean weight of 500 seeds from the transgenic line was higher, at 8.54mg, than that of WT seeds (8.1mg) (Fig. 2D).

### Seeds of *IgASE1* transgenic plants were less dormant than the WT

Seeds from the transgenic and WT plants were harvested and allowed to dry on a laboratory bench for 5, 10, and 15 days. They were then germinated on ½ MS agar plates without stratification and the percentage of seeds germinated after 48h were recorded. The germination rate of the transgenic seeds was 57, 98, and 100% compared to 25, 35, and 78%, respectively, for the WT seeds (Fig. 2E). Near 100% germination was found for stratified seeds of both WT and transgenic plants at all times (data not shown). Therefore, the germination rate of the transgenic seeds was higher than that of the WT seeds, implying that seeds from the *IgASE1*-expressing plants were less dormant and germinated sooner than the WT seeds.

### *35S:IgASE1* transgenic *Arabidopsis* was hypersensitive to ABA during germination and post-germination growth

**Fig. 3.**
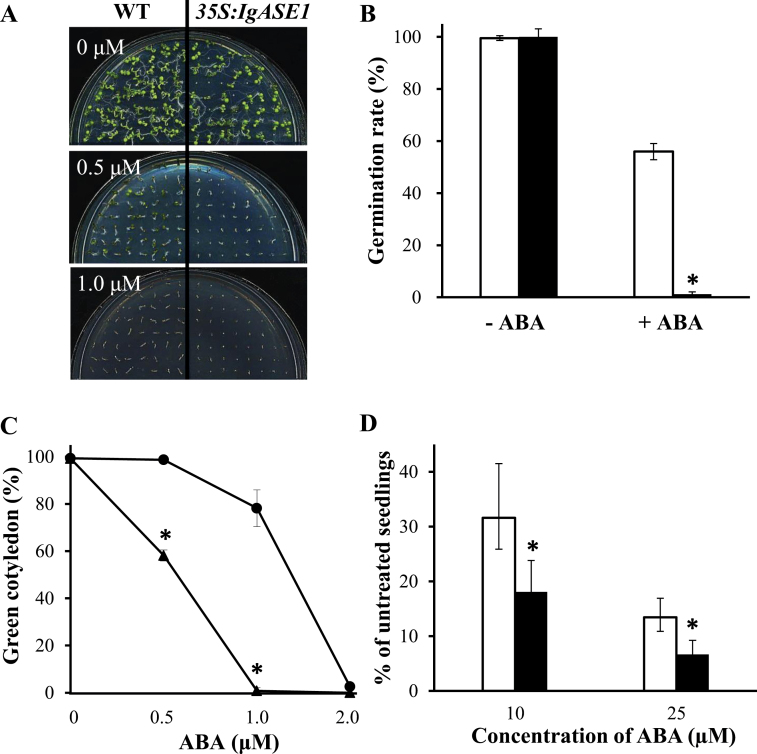
Effect of exogenous ABA on seed germination and early seedling development. (A) Effect of ABA on early seedling growth. Sterilized and stratified seeds were grown on ½ MS medium without or with 0.5 and 1 μM ABA for 8 days. (B) Percentage seed germination after 48h incubation of WT (white columns) and transgenic *Arabidopsis* (black columns) on ½ MS medium with or without 0.1 μM ABA. Values are means ± SE from three repeats; *n* = 56 for both WT and transgenic plants. (C) Percentage of seedlings that developed green cotyledons 12 days after sowing on media in the presence of different concentrations of ABA; WT, black circles; transgenic, black triangles. Values are the mean percentage ± SE (*n* = 52) from three repeats. *P < 0.05 in a Student’s *t* test. (D) Percentage fresh weight of the shoots of WT (white columns) and transgenic (black columns) plants relative to the untreated seedlings grown on ½ MS medium for 18 days without or with 10 and 25 μM ABA. Values are mean weight ± SE (*n* = 15) from three repeats. *P < 0.05 in a Student’s *t* test. This figure is available in colour at *JXB* online.

To test the sensitivity of transgenic *Arabidopsis* to ABA, germination and early seedling growth of the WT and the transgenic *Arabidopsis* plants were observed on ½ MS medium without ABA, or supplemented with different concentrations of ABA between 0 and 2 μM. Both germination and seedling growth of the WT and the transgenics were markedly inhibited as the ABA concentration increased (Fig. 3A). In the medium with 0.1 μM ABA, only about 1.2% of the transgenic seeds germinated whereas 56% of the WT seeds germinated after 48h (Fig. 3B). Thus germination of *35S:IgASE1* transgenic seeds was significantly more inhibited by ABA than for WT seeds, indicating the transgenic seeds were hypersensitive to ABA during germination.

The effect of ABA on post-germination growth of seedlings was analysed by scoring the percentage of germinated seeds that could form green cotyledons in the presence of different concentrations of ABA (Chen and Jones, 2004). Cotyledon opening and greening of both WT and transgenic plants were significantly inhibited as the ABA concentration increased (Fig. 3C). At 1 μM ABA almost all the transgenic cotyledons failed to turn green (as compared with those grown without ABA) while 78% of WT seedlings developed green cotyledons. Thus *IgASE1*-expressing seedlings were hypersensitive to ABA during post-germination growth.

The same trend was also observed for growth inhibition of seedlings during early development (Fig. 3D, Supplementary Fig. S1 available at *JXB* online). Five-day-old ½ MS-grown seedlings were transferred to fresh medium supplemented with 10 and 25 μM ABA (controls were transferred to fresh ½ MS medium without ABA). After 18 days, growth of both the transgenic and WT seedlings was much reduced by ABA compared to their untreated controls. However, ABA had a much greater effect on the transgenic than on the WT seedlings because while only 18.1 and 6.8% of the weights were retained in the 10 and 25 μM ABA-treated transgenic seedlings, that of the WT was nearly double that of the transgenic plants at 31.6 and 13.4% under the same ABA concentrations. Therefore, the growth of transgenic seedlings was reduced much more by ABA than that of WT seedlings, indicating that the *IgASE1-*expressing seedlings were hypersensitive to ABA during early seedling development.

### Restricted transpiration of transgenic plants was associated with increased foliar ABA levels

**Fig. 4.**
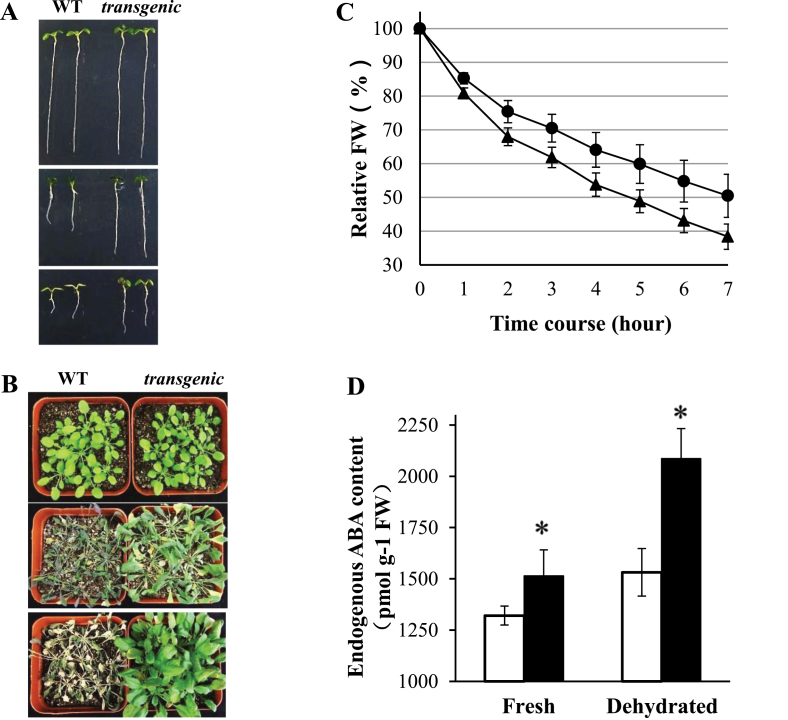
Response of WT and transgenic *Arabidopsis* plants to dehydration stress. (A) Response of seedlings to dehydration by mannitol and glycerol. Three-day-old ½ MS-grown seedlings were transferred to fresh ½ MS (top), ½ MS containing 300mM mannitol (middle), or 3% (v/v) glycerol (bottom) and grown for a further 7 days. (B) Drought treatment of soil-grown plants. 3-week-old plants (top) had water withheld for 22 days (middle) and were subsequently re-watered (bottom). (C) Water loss of detached leaves. Relative fresh weight (FW) is the percentage fresh weight of a leaf at a dry time point relative to its fresh weight at time 0. WT, black triangles; transgenics, black circles. The experiment was repeated three times and the data were averaged; values are means ± SE from five leaves for each experiment. (D) Endogenous ABA levels of the WT (white columns) and *35S:IgASE1* transgenic plants (black columns) estimated from untreated (Fresh) and 1-h air-dried (dehydrated) leaves. ABA concentration is expressed as pmol per gram of fresh weight. *P < 0.05 in a Student’s *t* test. This figure is available in colour at *JXB* online.

Since *IgASE1*-expressing transgenic *Arabidopsis* were hypersensitive to ABA treatment during seed germination and early seedling development, drought was mimicked by treating 3-day-old WT and transgenic seedlings with glycerol or mannitol for 7 days. Both mannitol and glycerol inhibited root growth of both the WT and transgenic plants, but root growth was significantly more inhibited in WT than transgenic seedlings (Fig. 4A). These results indicate that the transgenic *Arabidopsis* exhibited greater osmotic tolerance.

Furthermore, 3-week-old plants were subjected to drought stress by withholding water for 22 days. Both WT and transgenic plants were withered at the end of the treatment. However, when these plants were drenched with water most of the transgenic plants quickly recovered and continued to grow to maturity while none of the WT plants survived under these conditions (Fig. 4B), indicating that the transgenic plants are more drought-resistant than the WT ones.

To test whether these responses could result, at least in part, from lower transpiration rates of transgenic plants, detached leaf water loss was measured over time. Relative fresh weight of the detached leaves of *IgASE1*-expressing transgenics was significantly higher than that of the WT leaves throughout the experiment. After 7h the transgenic leaves retained 50.5% of their original weight whereas only 38.4% of the original weight of the WT leaves was retained (Fig. 4C).

Endogenous foliar ABA levels in both fresh leaves (immediately detached) and leaves dehydrated for 1h were higher in transgenic plants compared to WT plants (Fig. 4D). This suggests that ABA production was elevated in the transgenic plants in both normal and dehydration conditions.

**Fig. 5.**
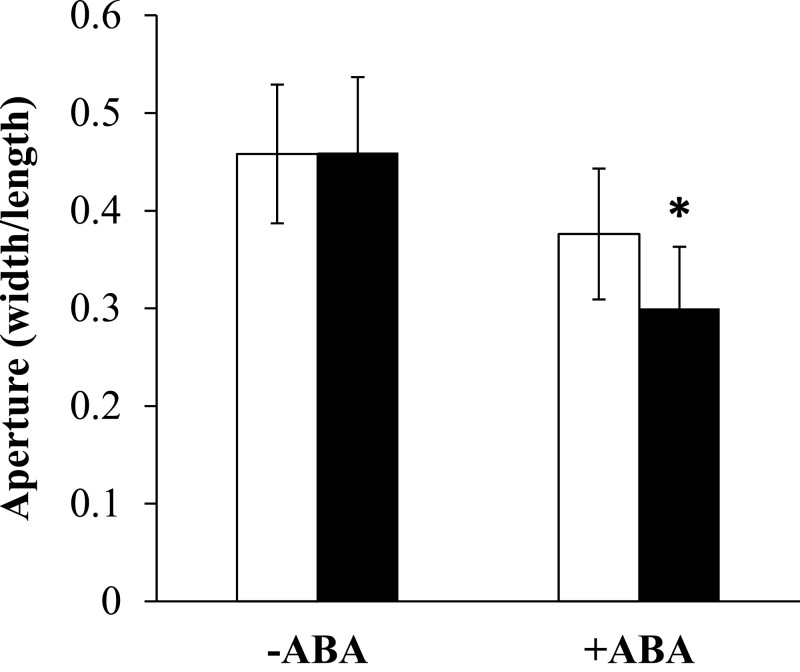
ABA-induced stomatal closure in WT (white columns) and *35S:IgASE1* transgenic plants (black columns). Data represent the mean ± SE from three independent experiments (*n* = 50–60 per experiment). *P < 0.05 in a Student’s *t* test.

Because water loss is regulated via stomata, the stomatal apertures of transgenic and WT plants were compared in the presence and absence of ABA. While both the *35S:IgASE1* transgenic leaves and the WT leaves had similar stomatal apertures without ABA treatment (7.7±0.6 µm in width and 16.8±1.4 µm in length for WT, 8.2±0.7 µm width and 18.0±0.5 µm length for the transgenics), the aperture of the stomata of the transgenic plants was smaller than that of the WT plants when ABA was present (6.0±0.7 µm width and 15.9±1.4 µm length for WT, 4.8±0.3 µm width and 15.9±1.3 µm length for the transgenic plants; Fig. 5), suggesting that *35S:IgASE1* transgenic leaves were more sensitive to ABA-induced stomatal closure than the WT leaves.

These combined data indicate that the drought resistance of *IgASE1*-expressing plants is regulated via the ABA-mediated stress-response pathway.

### Exogenous application of EDA and ETrA can mimic ABA and drought responses in WT plants, similar to that found in transgenic plants

**Fig. 6.**
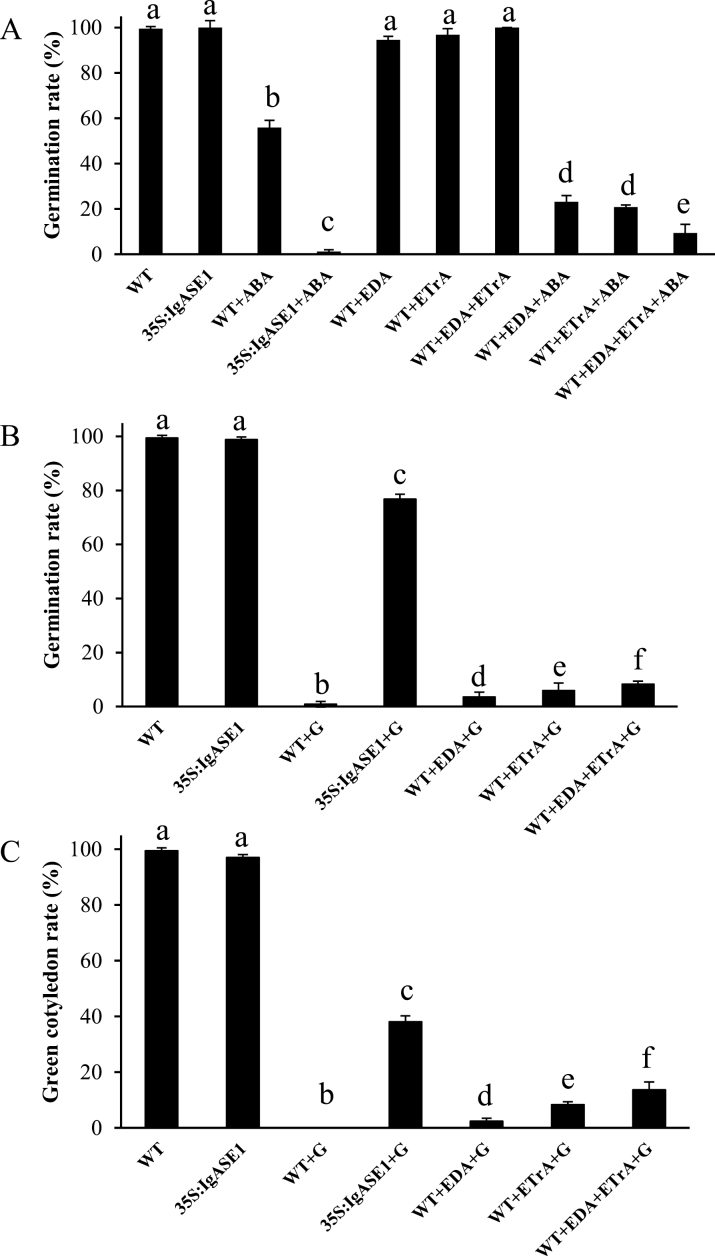
The effect of exogenous application of EDA and ETrA on seed germination and seedling establishment. (A) Germination of WT and *35S:IgASE1* transgenic *Arabidopsis* on ½ MS media with or without ABA (0.1 μM), EDA (10 µM), ETrA (10 µM), or EDA+ETrA (10 µM each) after 48h. (B) Germination of WT and *35S:IgASE1* transgenic plants on ½ MS media without or with glycerol (G; 2%), EDA (10 µM), ETrA (10 µM), or both (10 µM each) after 48h. (C) Quantitative analysis of the percentage of greening cotyledons on WT and transgenic plants grown on media without or with glycerol (2%), EDA (10 µM), ETrA (10 µM), or both (10 µM each) after 7 days. Different letters indicate statistically different values after one-way ANOVA.

To determine whether the hypersensitivity to ABA in the transgenic plants was caused by the newly synthesized C_20_ VLCPUFAs EDA and ETrA in these plants, exogenous application treatments with these two fatty acids were performed on WT plants during germination. Germination rates of both WT and transgenic seeds were close to 100% and were not significantly altered by exogenous application of EDA, ETrA, or EDA+ETrA to the germination media (Fig. 6A). However, in the presence of 0.1 μM ABA the germination rate of WT seeds was reduced to nearly 56%. This inhibitory effect was enhanced by exogenous supplementation of EDA, ETrA, or EDA+ETrA, under which treatments only 23.2, 20.8, and 9.5% of the seeds germinated, respectively (Fig. 6A). Next, different amounts of EDA+ETrA were added to the germination media. Although 92% of the untreated seeds germinated after 48h, only 47% germinated when 0.5 µM ABA was present (Supplementary Fig. S2 available at *JXB* online). This reduction in seed germination by ABA was significantly enhanced by adding 2.5, 5, and 10 μM of each of EDA and ETrA in a dose-dependent manner, where only 32.5, 7.6, and 6.0% of the seeds germinated, respectively (Supplementary Fig. S2 available at *JXB* online). Although this was higher than the ABA-treated transgenic seeds, of which only 1.2% germinated, it suggests that the increased sensitivity to ABA of *IgASE1*-expressing plants is most likely due to the endogenous EDA and ETrA or their derived metabolites in the transgenic plants.

Experiments then determined whether the drought resistance in the transgenic *Arabidopsis* was caused by endogenous EDA and ETrA, by germinating WT seeds in media containing these two fatty acids coupled with 2% glycerol to mimic dehydration conditions. When glycerol was present, fewer than 1% of the WT seeds germinated whereas 77% of the transgenic seeds germinated. The inhibitory effect of glycerol on WT seed germination was alleviated by supplementing the media with EDA and ETrA, for which 3.6, 6.0, and 8.3% germinated with EDA, ETrA, or EDA+ETrA present, respectively (Fig. 6B).

The percentage of seedlings that developed green cotyledons on glycerol-containing media was 38% for the *35S:IgASE1* transgenic plants, while no WT seedlings were recovered. However, when EDA, ETrA, or both EDA and ETrA were supplemented in the presence of glycerol, the numbers of WT seedlings bearing green cotyledons increased to 2.4, 8.3, and 13.7% for EDA, ETrA or EDA+ETrA, respectively (Fig. 6C). Thus EDA and ETrA can partially alleviate the inhibitory effects of glycerol on seed germination and early seedling development in the WT. Thus responses of *35S:IgASE1* transgenic *Arabidopsis* plants are due, at least in part, to these two newly synthesized C_20_ VLCPUFAs or their derived metabolites.

### Transcript levels of ABA biosynthetic and catabolic genes were affected in transgenic *Arabidopsis* under stress conditions

**Fig. 7.**
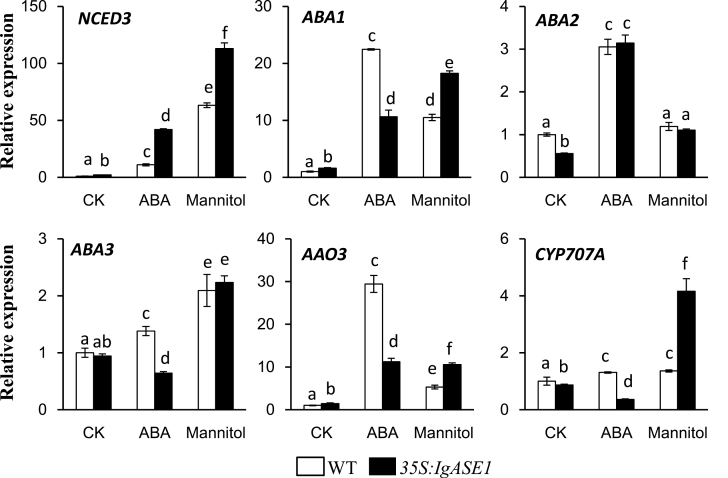
Transcript levels of genes involved in ABA metabolic pathways. Transcript levels were measured by quantitative RT-PCR from untreated solvent control (CK), ABA (100 µM), and mannitol (300mM)-treated 7-day-old seedlings. Experiments were performed three times. Each bar represents the mean ± SE (*n* = 3). Different letters indicate statistically different values after one-way ANOVA.

To determine whether foliar ABA accumulation of *35S:IgASE1* transgenics could be explained by changes in gene expression, the expression profiles of some ABA biosynthetic genes, such as 9-cis-epoxycarotenoid dioxygenase 3 (*NCED3*), *ABA1*, *ABA2*, *ABA3*, and abscisic aldehyde oxidase 3 (*AAO3*), as well as the ABA catabolic gene *CYP707A*, were determined by quantitative RT-PCR (Fig. 7). Samples were analysed following 3h ABA (100 µM) or mannitol (300mM) treatment in 7-day-old plate-grown seedlings.

In the untreated control seedlings, mRNA levels of *NCED3* and *ABA1* were much higher while those of *ABA2* and *CYP707A* were much lower in the transgenic plants than in the WT, although those of *ABA3* and *AAO3* were similar. Upon treatment with mannitol, *NCED3*, *ABA1*, *ABA3*, and *AAO3* transcripts were upregulated in both the WT and transgenic plants and the expression levels of *NCED3* (1.8-fold), *ABA1* (1.7-fold), *ABA2* (2.0-fold), and *AAO3* (2.0-fold) were much higher in the *35S:IgASE1* transgenic seedlings than in the WT, while expression of *ABA3* was similar between WT and transgenic plants (Fig. 7). However, expression of *CYP707A* in the *35S:IgASE1* transgenic *Arabidopsis* was also 3.1-fold higher than in WT plants. These combined results indicate that genes regulating ABA turnover were more active in the transgenic *Arabidopsis* under dehydration stress.

In ABA-treated seedlings, mRNA levels of *NCED3*, *ABA1*, *ABA2*, and *AAO3* were increased in both the WT and the transgenic plants. However, among them only *NCED3* was expressed at much higher levels in the transgenics than in the WT. Levels of *ABA1*, *ABA3*, and *AAO3* mRNAs in the transgenic plants were lower than those in the WT while *ABA2* mRNA levels were similar in both sets of plants. Interestingly, ABA inhibited the expression of *CYP707A* in the transgenic plants (Fig. 7). This suggests that the inhibitory effect of exogenous ABA during seedling growth of the *IgASE1* transgenic plants (Fig. 3C, D) may be enhanced by the upregulation of *NCED3* coupled with less ABA being degraded by the less-active *CYP707A*, leading to an overall higher level of endogenous ABA being produced in the transgenic compared to the WT *Arabidopsis*.

### Expression levels of ABA receptor and signalling genes were altered in the transgenic *Arabidopsis*

**Fig. 8.**
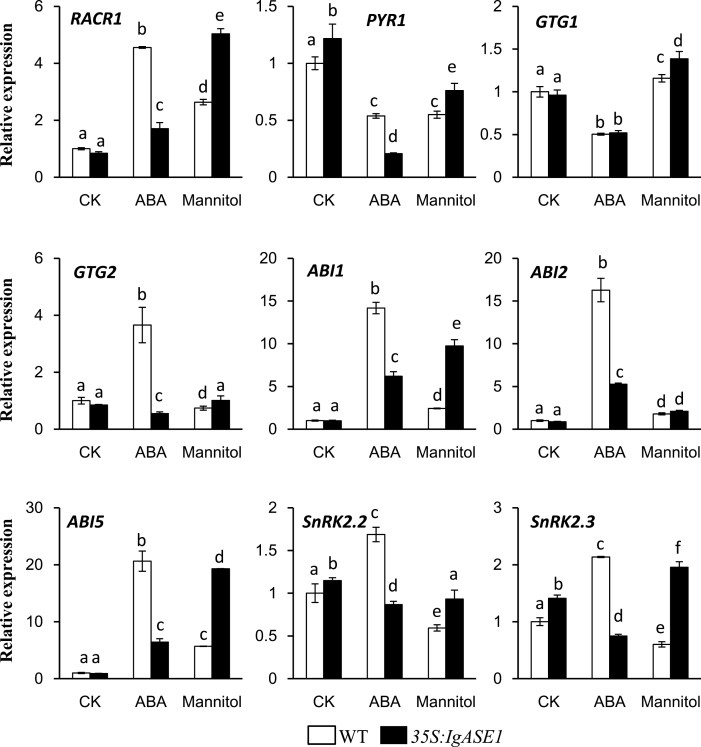
Expression levels of genes involved in ABA signalling pathways. Transcript levels were measured by quantitative RT-PCR from untreated solvent control (CK), ABA (100 µM), and mannitol (300mM)-treated 7-day old seedlings. Experiments were performed three times. Each bar represents the mean ± SE (*n* = 3). Different letters indicate statistically different values after one-way ANOVA.

The ABA receptor genes *RCAR1*, *PYR1*, *GTG1*, and *GTG2* as well as signalling-related genes (*ABI1*, *ABI2*, *ABI5*, *SnRK2.2*, and *SnRK2.3*) were also monitored in the untreated and ABA- or mannitol-treated seedlings. mRNA levels of most of the tested genes were similar in the control seedlings apart from *PYR* and *SnRK2.3*, which were slightly upregulated in the transgenic plants compared to the WT (Fig. 8). However, mannitol strongly upregulated the expression of *RACR1*, *ABI1*, and *ABI5* in both the WT and the transgenic seedlings; this was more so in the transgenic than in the WT seedlings. Of the four ABA receptor genes studied here, only *RACR1* was significantly upregulated by mannitol and its expression level was 1.9-fold higher in the transgenic plants than in the WT, while the expression of *PYR1* was decreased, *GTG2* was similar, and *GTG1* was only modestly upregulated. The signalling-related genes *ABI1* and *ABI5* were also significantly upregulated while *ABI2* was only slightly upregultaed in both the WT and transgenic seedlings and the mRNA levels of *ABI1* and *ABI5* were 4.0- and 3.4-fold higher in the transgenic plants than in WT, respectively. *SnRK2.2* and *SnRK2.3* were decreased in the WT while *SnRK2.2* was similarly expressed and *SnRK2.3* was slightly upregulated in the transgenic compared to the WT plants (Fig. 8). These results imply that the drought resistance of the *35S:IgASE1* transgenic plants was at least partly due to the upregulation of genes in the ABA-mediated drought stress-response pathway. ABA, on the other hand, seems to have less effect on the transcript levels of these genes in the transgenic plants than in the WT (Fig. 8).

### Regulation of ABA-dependent and -independent drought-response genes in transgenic plants

**Fig. 9.**
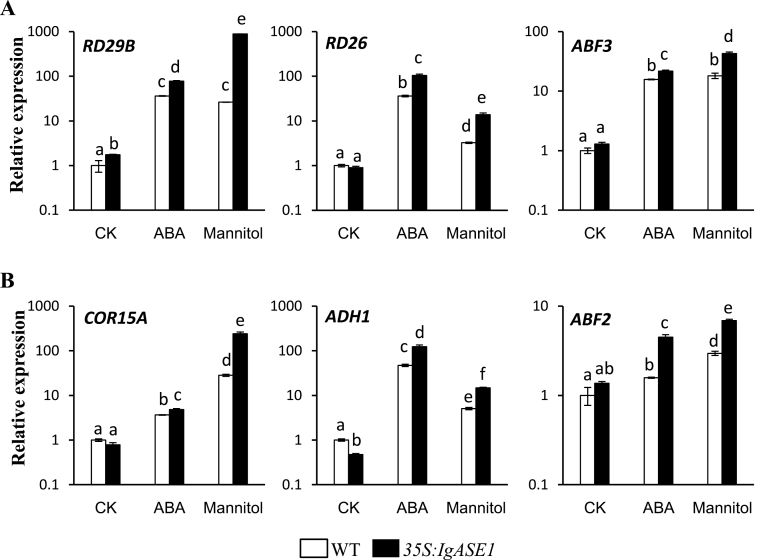
Expression levels of genes involved in the ABA-dependent and ABA-independent pathways. Transcript levels were measured by quantitative RT-PCR from untreated solvent control (CK), ABA (100 µM), and mannitol (300mM)-treated 7-day old seedlings. (A) Transcript levels of the three ABA-dependent genes. (B) Transcript levels of the three ABA-independent genes. Experiments were performed three times. Each bar represents the mean ± SE (*n* = 3). Different letters indicate statistically different values after one-way ANOVA.

To determine the involvement of EDA and ETrA in the drought response, and whether its role was limited to the ABA-regulated pathway, expression levels of several drought-inducible genes that display different sensitivity to exogenously applied ABA (Ding *et al.*, 2011) were assayed. The ABA-dependent genes *RD29B*, *RD26*, and *ABF3* were strongly induced by both exogenous ABA and mannitol (Fig. 9A) in both *IgASE1*-expressing and WT plants. However, their expression levels were much higher in the *35S:IgASE1* transgenic plants than in the WT.

We next monitored the expression of three ABA-independent genes, *COR15A*, *ADH1*, and *ABF2*. The transcript levels of *COR15A* and *ABF2* were similar between the WT and transgenic plants while *ADH1* was much lower in the transgenic than the WT plants in the untreated control samples. Followed ABA treatment the transcript levels of *COR15A*, *ABF2*, and *ADH1* were increased by 3.6- and 6.2-, 47.2- and 261.5-, and 1.6- and 3.3-fold for the WT and transgenic plants, respectively. Mannitol had similar effect where the transcript levels were increased by 28.1- and 307.2-fold for *COR15A*, 5.1- and 31.4-fold for *ADH1*, and 3.0- and 5.0-fold for *ABF2* in WT and transgenic plants, respectively. Therefore, both ABA and mannitol can strongly induce the expression of all three genes and their transcript levels were much higher in the *35S:IgASE1* transgenic plants than in the WT (Fig. 9B).

## Discussion

Fatty acids and their derived metabolites are not only major structural components of the cell membrane, but also function as modulators of a multitude of signal transduction pathways evoked by environmental and developmental changes. Therefore, remodelling the fatty acid composition of an organism can alter metabolism as well as the complex interaction array of signalling cascades that affect a range of physiological responses (Savchenko *et al.*, 2010). While studies show that the VLCPUFAs, such as ARA and other eicosapolyenoic acids, play important roles during biotic stress in plants (Bostock *et al.*, 2011), hitherto there have been no reports of their effects on abiotic stress in plants.

We utilized a transgenic *Arabidopsis* line expressing a fatty acid elongase gene from *I. galbana* that was specific for the two C_18_ Δ^9^ long-chain unsaturated fatty acids LA and ALA (Qi *et al.*, 2002). This transgenic line can produce EDA and ETrA to 25 mol% of total leaf fatty acids. Effects of these transgene products on plant growth and development and the modulation of ABA in relation to seed germination, seedling establishment, and drought resistance in young vegetative plants were assayed. Phenotypic differences between this transgenic line and WT *Arabidopsis* were detected in leaves, flowers, and seed size (Fig. 2). While a previous study with a different transgenic line that contained less EDA and ETrA (15%) reported that the transgenics were morphologically indistinguishable from the WT plants (Fraser *et al.*, 2004), differences in morphology between the two transgenic lines could be the direct result of different amounts of EDA and ETrA accumulated. *Arabidopsis* plants that overexpress Fatty Acid Elongase 1 (FAE1) under the control of the CaMV35S promoter showed dramatic phenotypic alterations (Millar *et al.*, 1998). FAE1 is a seed-specific fatty acid elongase that catalyses the production of C_20_+ very-long-chain saturated and mono-unsaturated fatty acids (VLCFAs) in *Arabidopsis* seed oil (O’Neill *et al.*, 2003). WT *Arabidopsis* leaves contain less than 1% of these VLCFAs, which are mainly present in the epidermal cuticular waxes (Millar and Kunst, 1997). However, the *CaMV35S:FAE1* transgenic *Arabidopsis* plants can accumulate high levels (>30%) of VLCFAs in their leaf membrane lipids. Although the transgenic plants with relatively low levels of VLCFAs appeared to be like the WT, the transgenic plants with high levels of VLCFAs exhibited a wide range of morphological changes and some even failed to survive (Millar *et al.*, 1998; Bach *et al.*, 2008). Recent studies show that VLCFAs have a wide range of physiological and structural functions and are crucial for many biological processes such as cell division and expansion, cell proliferation, or differentiation (Bach *et al.*, 2011). High levels of VLCPUFAs also had similar, although reduced, effects on plant growth and development (Fig. 2), which suggests that they may function in the same pathways as VLCFAs.

Seeds of the transgenic plants displayed lower levels of dormancy than the WT (Fig. 2E). When first harvested, seeds are usually highly dormant and this dormancy is gradually reduced during dry storage to allow germination. It is well established that the plant hormone ABA plays an essential role in the induction of seed dormancy and inhibition of germination (Carrera *et al.*, 2008; Finkelstein *et al.*, 2008). In line with this, germination of the transgenic seeds was strongly inhibited by ABA (Fig. 3A). ABA also inhibited early seedling establishment and growth (Fig. 3A–C), indicating that the accumulation or perception of ABA might be different in the transgenics compared to the WT during seed maturity, germination and post-germination periods. The fact that these effects could be altered by exogenous application of EDA and ETrA to the WT (Fig. 6, Supplementary Fig. S2 available at *JXB* online) indicates the direct involvement of these two eicosapolyenoic acids in the regulation of ABA-mediated seed germination and early seedling development. Further, and perhaps most importantly, adult *35S:IgASE1* transgenic plants were apparently more drought resistant than the WT (Fig. 4). This is likely due to their smaller leaf area and lower transpiration rate of the transgenic plants (Figs 2A, B and 4C). They contained more endogenous ABA than the WT and their stomata more readily closed upon ABA treatment (Figs 4D and 5). Since inhibitory effects of mannitol and glycerol on seed germination and seedling growth were reduced by EDA and ETrA, it suggests that these two fatty acids played positive roles in regulating drought response in the transgenic *Arabidopsis* (Fig. 6).

ABA biosynthesis and degradation contribute to ABA homeostasis during plant development and stress conditions. To determine whether the overall production of ABA in the transgenic plants was different from the WT the endogenous ABA levels were measured. This revealed that the transgenic plants contained more ABA in their leaves; this was more so under dehydration conditions (Fig. 4D). This indicates that the drought resistance in the *35S:IgASE1* transgenic *Arabidopsis* is involved in the accumulation and/or perception of ABA. Indeed, the transcript levels of ABA biosynthesis and signalling genes were very different in the control untreated as well as osmotic stress-/ABA-treated transgenic seedlings compared to WT (Figs 7 and 8). Although *ABA2*, *ABA3*, and *AAO3* transcript levels were lower and similar respectively in the transgenics compared to the WT, the expression of *ABA1* and *NCED3* were significantly higher in the *35S:IgASE1* transgenic seedlings (Fig. 7). Further, the transcript level of the major enzyme for ABA catabolism, *CYP707A*, was much lower in the *35S:IgASE1* transgenic plants than in WT. This suggests that the higher foliar ABA level in the transgenic plants was due to the combined action of higher activity of ABA1 and NCED3 for biosynthesis and lower CYP707A activity for ABA degradation, resulting in overall higher ABA content in the transgenics than in the WT in untreated plants. Upon treatment with mannitol the transcript levels of four out of the five ABA biosynthetic genes (apart from *ABA2* in the WT) as well as the catabolic gene *CYP707A* were all significantly upregulated, more so in the transgenics than in the WT (Fig. 7). The increase in expression of CYP707A during osmotic stress can aid the plants to rapidly degrade the excess ABA generated; therefore, the homeostatic ABA levels can be maintained (Kushiro *et al.*, 2004).

Recent studies identified and confirmed the PYR/RCAR proteins as ABA receptors that interact with the protein phosphatase 2Cs (PP2Cs), such as ABI1 and ABI2, which function as negative regulators of ABA signalling (Ma *et al.*, 2009; Park *et al.*, 2009). In contrast, SnRK2s act as positive regulators of downstream signalling (Vlad *et al.*, 2009). Thus, in the absence of ABA, the PP2Cs are active and repress SnRK2 activity and downstream signalling. In the presence of ABA, PYR/RCARs interact with PP2Cs and inhibit phosphatase activity, allowing SnRK2 activation and phosphorylation of target proteins (Hubbard *et al.*, 2010). Enhancing ABA signalling through PYR/PYR1-LIKE (PYL)/RCAR ABA receptors has been shown to improve plant drought resistance. For example, transgenic *Arabidopsis* plants overexpressing ABA receptor PYL4, particularly its mutated variant PYL4 A194T, exhibited an enhanced response to ABA and drought resistance via ABA-dependent inhibition of PP2Cs (Pizzio *et al.*, 2013). The *35S:IgASE1* transgenic *Arabidopsis* were hypersensitive to ABA during seed germination and early seedling development (Fig. 3). This could be explained by the higher transcript levels of *PYR1*, *SnRK2.2,* and *SnRK2.3* in the transgenics than WT (Fig. 8). Upon treatment with mannitol only *RCAR1* and *GTG1* of the four ABA receptor genes assayed were significantly upregulated. The signalling-related genes, such as *ABI1*, *ABI2*, and *ABI5*, were also significantly upregulated in both WT and *35S:IgASE1* transgenic plants, and higher levels of expression of these genes were found in the transgenics compared to WT (Fig. 8). Therefore, our quantitative RT-PCR data demonstrated that ABA signalling was also modified by upregulating receptor genes and signalling-related genes in the transgenic plants under osmotic stress condition (Fig. 8). However, exogenous application of ABA had the opposite effect on gene expression of *RACR1*, *ABI1*, *ABI2*, *ABI5*, *SnRK2.2*, and *SnRK2.3* where lower levels of transcripts were found in transgenic seedlings compared to WT (Fig. 8). In addition, we also found that the ABA-dependent (*RD29B*, *RD26*, and *ABF3*) as well as the ABA-independent (*COR15A*, *ADH1*, and *ABF2*) drought-inducible genes displayed increased transcript levels in the transgenics following both ABA and osmotic stress (Fig. 9A, B), implying that stress adaptation in the EDA- and ETrA-producing transgenic *Arabidopsis* was complex, involving both ABA-dependent and -independent pathways.

It is thought that plant drought tolerance is largely dependent on the inherent level of fatty acid unsaturation and/or the ability to maintain or adjust fatty acid unsaturation (Mikami and Murata 2003). For example, water deficit decreased ALA (18:3) and LA (18:2) concentrations in *Brassica napus* (Dakhma *et al.*, 1995). Relatively higher proportions and levels of unsaturated fatty acids, particularly 18:3 and 18:2, are associated with leaf dehydration tolerance and post-drought rehydration in Kentucky bluegrass (Xu *et al.*, 2011). Overexpression of the *Arabidopsis* FAD3 and FAD8 ω-3 desaturases in tobacco cells and plants increases the ratio of 18:3 to 18:2, and increases cellular resistance to osmotic stress and whole-plant drought tolerance (Zhang *et al.*, 2005). The major polyunsaturated fatty acids in *Arabidopsis* leaves are 18:3 (43.4%) and 18:2 (17.7%) (Fig. 1B, C). In the *35S:IgASE1* transgenic *Arabidopsis*, although the amounts of both 18:2 and 18:3 are significantly reduced due to the production of the two C_20_ polyunsaturated fatty acids 20:2 and 20:3 (Fig. 1B, C), the plant exhibited enhanced drought and osmotic resistance (Fig. 4). This suggests that the two newly synthesized C_20_ polyunsaturated fatty acids EDA and ETrA play a positive role in the drought stress response that is similar to 18:2 and 18:3; hence, the transgenic plants were not compromised by the replacement of the two C_18_ polyunsaturated fatty acids with EDA and ETrA. Instead, the enrichment of EDA and ETrA in the membrane phospholipids phosphatidylcholine, phosphatidylethanolamine, phosphatidate, and phosphatidylinositol (Fraser *et al.*, 2004) may have helped to maintain membrane structure and fluidity. This is important for membrane integrity and the functionality of integral membrane proteins, including ABA-biosynthetic and signalling machinery proteins.

Although the exact mechanism of the direct involvement of the two eicosapolyenoic acids EDA and ETrA in ABA metabolism and signalling is unknown, we nevertheless provide evidence that they can stimulate the expression of ABA biosynthesis genes under osmotic stress. They also control the expression of stress regulation and ABA-responsive genes. Through these combined actions, they may enhance the metabolism and responsiveness to ABA of the plant cell, therefore helping to maintain water balance by decreasing water loss through stomatal closure and hence improving drought resistance in these transgenic plants.

Here we show that eicosapolyenoic acids such as EDA and ETrA are involved in a range of activities during the life cycle of *Arabidopsis* as well as in the adaptation of plants to abiotic stress such as drought, by regulating ABA-dependent and -independent pathways.

## Supplementary material

Supplementary material is available at *JXB* online.

Table S1. Sequences of primers used in this study.

Figure S1. *IgASE1*-expressing seedlings (*35:IgASE1*) were more sensitive to ABA during early seedling development. WT and transgenic seeds were germinated on ½ MS medium for 5 days. They were then transferred to ½ MS medium supplemented without (0 µM) or with 10 and 25 µM ABA. They were scanned after 18 days culture in an environmental controlled growth room under long-day-length conditions.

Figure S2. Effect of different amounts of exogenously supplied fatty acids to percentage of germinated seeds of the WT *Arabidopsis* in the presence of 0.5 μM ABA. Seeds were germinated for 48h. FA1, 2.5 μM of each EDA and ETrA; FA2, 5 μM of each EDA and ETrA; FA3, 10 μM of each EDA and ETrA. Different letters indicate statistically different values after one-way analysis of variance.

Supplementary Data

## Abbreviations:

ABA: abscisic acid
ALA: α-linolenic acid
ARA: arachidonic acid
EDA: eicosadienoic acid
EtrA: eicosatrienoic acid
LA: linoleic acid
MS: Murashige and Skoog
PP2C: protein phosphatase 2C
VLCFA: very-long-chain saturated and mono-unsaturated fatty acid
VLCPUFA: very-long-chain polyunsaturated fatty acid
WT: wild-type.
