# Supplementary material for: Accumulation of eicosapolyenoic acids enhances sensitivity to abscisic acid and mitigates the effects of drought in transgenic Arabidopsis thaliana
Source: J Exp Bot. 2014 Mar 7;65(6):1637–49. doi: 10.1093/jxb/eru031 (PMC3967093; doi:10.1093/jxb/eru031)
Supplement: Supplementary Data [file supp_65_6_1637__index.html]

Accumulation of eicosapolyenoic acids enhances sensitivity to abscisic acid and mitigates the effects of drought in transgenic Arabidopsis thaliana — Accumulation of eicosapolyenoic acids enhances sensitivity to abscisic acid and mitigates the effects of drought in transgenic Arabidopsis thaliana — Supplementary Data 

# Accumulation of eicosapolyenoic acids enhances sensitivity to abscisic acid and mitigates the effects of drought in transgenic *Arabidopsis thaliana*

## Supplementary Data

Data files

**Files in this Data Supplement:**

- Supplementary Data - Supplementary Data
