# Supplementary material for: Accumulation of eicosapolyenoic acids enhances sensitivity to abscisic acid and mitigates the effects of drought in transgenic Arabidopsis thaliana
Source: J Exp Bot. 2014 Mar 7;65(6):1637–49. doi: 10.1093/jxb/eru031 (PMC3967093; doi:10.1093/jxb/eru031)
Supplement: Supplementary Data [file supp_eru031_jexbot097782_file001.pdf]

Accumulation of eicosapolyenoic acids confers enhanced sensitivity to abscisic acid and mitigates the effects of drought in transgenic *Arabidopsis thaliana*

Xiaowei Yuan, Yaxiao Li, Shiyang Liu, Xinzheng Li, and Baoxiu Qi

Table 1S. Sequences of primers used in this study.

Primer	Sequence (5'-3')
ACT2-F	TTGTGCTGGATTCTGGTGATG
ACT2-R	CGCTCTGCTGTTGTGGTG
NCED3-F	ACAAGGTCGCAAGATTCGG
NCED3-R	TTGAGTCTGGTGGAGTCATACAG
ABA1-F	AATGGTCAACGCTATGAAGGT
ABA1-R	CCCGTGTAACAAGTGTAGCCT
ABA2-F	TGGAGGAGCCACAGGGATAG
ABA2-R	CTGCTTCGTGGTGAGTCCAA
ABA3-F	GGATCATGCTGGTTCTACTTTG
ABA3-R	TGTCGAGCATCCGCTATAAG
AAO3-F	AGCAGTTGTGAAGCCGTTAG
AAO3-R	ACCATACGCTTGTTGAATGAG
CYP707A-F	AGTCCATCAACACCCTCG
CYP707A-R	TACTTTCCTTAGTTCCTCGCT
RCAR1-F	CGAGACGGTGCAATACGT
RCAR1-R	AAGACTGCCGATTTCAGG
PYR1-F	TCCAGGAAGCTGTTCATCACT
PYR1-R	CGACGGAGCAGGATTTGA
GTG1-F	AGGCAAGGATGATAAACTGG
GTG1-R	GCTGAAGGCATAGGGAAAT
GTG2-F	GTGCGGTCGGTTCAAGAT
GTG2-R	CCACCTCTGCCTCCATTAG
ABI1-F	TCCCGTCTCACATCTTCGTC
ABI1-R	CTTCTCTATCCGGTTTATGGTCA
ABI2-F	TGTTCCATTCAGACCATTCAC
ABI2-R	ATATCCACACCTGCCATAGC
ABI5-F	TATGGGAGGGCTAAGGGG
ABI5-R	CGCATTCTCTTCTTTCAACTG
SNRK2.2-F	ATGGTCTTGCGGTGTAAC
SNRK2.2-R	GGTGTAAGTCCTCTGGGAT
SNRK2.3-F	GGTCACTAAGGCATCCTAATA
SNRK2.3-R	GCAAATCCGCTCGTAAAG
RD29B-F	GGGAAAGGACATGGTGAGG

RD29B-R	ACGGTGGTGCCAAGTGAT
RD26-F	TCCCAAAGGCACTAAAACCAA
RD26-R	GACACAACACCCAATCATCCA
ABF3-F	TTCAATGATGGGAAACAATACC
ABF3-R	ACTAATCGTCCGAGGCAAG
COR15A-F	AAGAGGCATTAGCAGATGGTGAG
COR15A-R	GTTTGCGGCTTCTTTTCCTTT
ADH1-F	CTTGGTGCTGTTGGTTTAGG
ADH1-R	TGTCATGGTCTTTCGGGTT
ABF2-F	TTAGGCGTTGGTGCTGTG
ABF2-R	TTGTTTGGTCTGCCGTGA

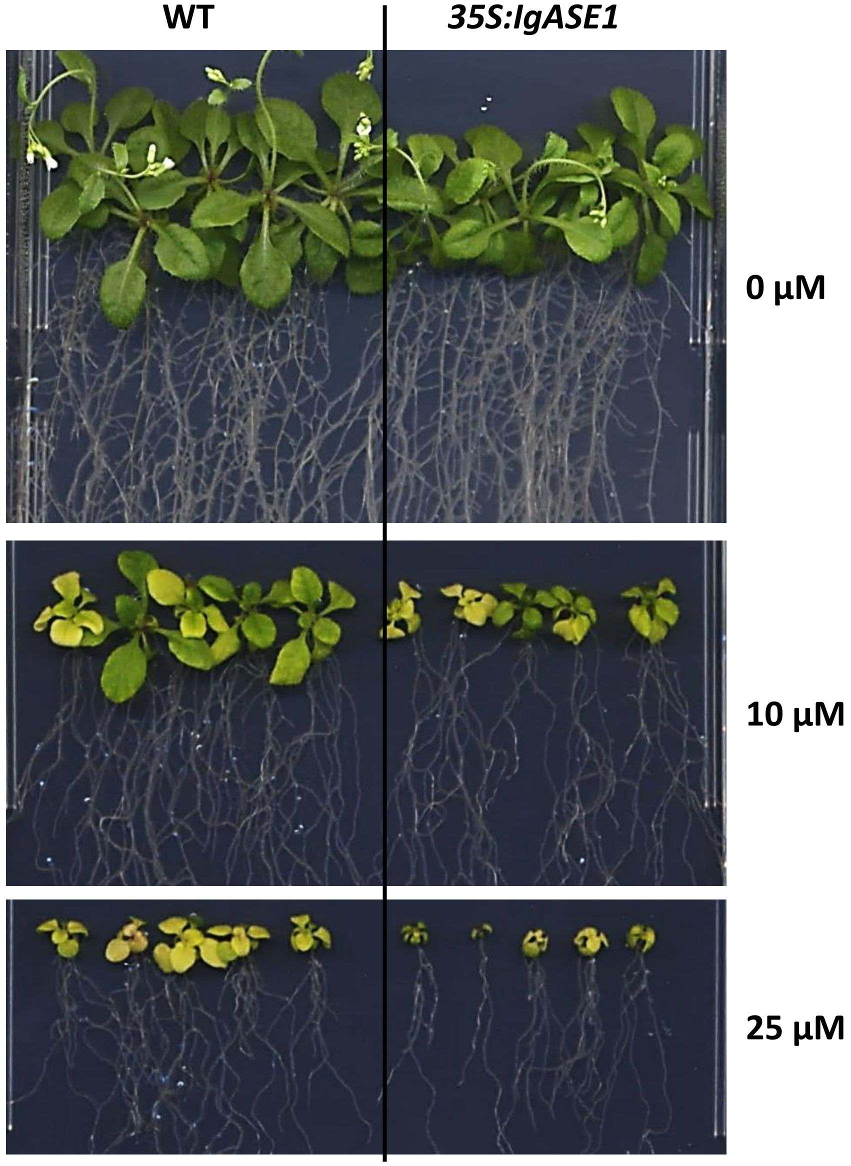

Fig. S1. *IgASE1* expressing seedlings (*35:IgASE1*) were more sensitive to ABA during early seedling development. WT and transgenic seeds were germinated on ½ MS media for 5 days. They were then transferred to ½ MS media supplemented without (0 µM), or with 10 and 25 µM ABA. They were scanned after 18 days cultavition in an environmental controlled growth room under long days.

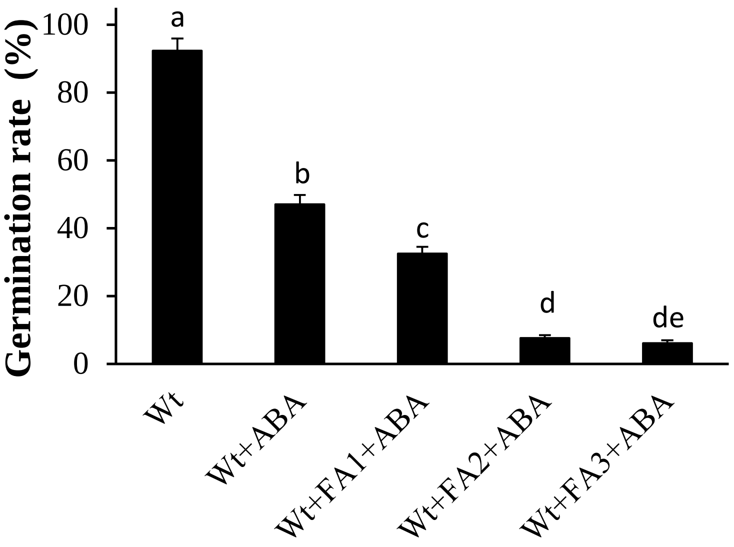

Fig. S2. Effect of different amounts of exogenously supplied fatty acids to percentage of germinated seeds of the wild-type Arabidopsis in the presence of 0.5 µM ABA. Seeds were germinated for 48h. FA1, 2.5µM of each EDA and ETrA; FA2, 5µM of each EDA and ETrA; FA3, 10µM of each EDA and ETrA). Different letters indicate statistically different values after one-way ANOVA.
